# Kinetic Study of Anaerobic Adhesive Curing on Copper and Iron Base Substrates

**DOI:** 10.3390/ma17122886

**Published:** 2024-06-13

**Authors:** Juana Abenojar, Sara López de Armentia, Juan Carlos del Real, Miguel Angel Martínez

**Affiliations:** 1 Materials Science and Engineering Department, IAAB, Universidad Carlos III de Madrid, 28911 Leganes, Spain; mamc@ing.uc3m.es; 2 Mechanical Engineering Department, Universidad Pontificia Comillas, 28015 Madrid, Spain; sara.lopez@comillas.edu (S.L.d.A.); delreal@comillas.edu (J.C.d.R.); 3 Institute for Research in Technology, Universidad Pontificia Comillas, 28015 Madrid, Spain

**Keywords:** anaerobic adhesives, curing rate, model-free kinetics, Kamal model, Kissinger model, torsion test

## Abstract

Anaerobic adhesives (AAs) cure at room temperature in oxygen-deprived spaces between metal substrates. The curing process is significantly influenced by the type of metal ions present. This study investigates the curing kinetics of a high-strength AA on iron and copper substrates using differential scanning calorimetry (DSC). The activation energy and kinetic parameters were determined with different empiric models, revealing that curing on copper is faster and more complete compared to iron. The findings suggest that copper ions lower the activation energy required for curing, enhancing the adhesive’s performance. This research addresses the gap in understanding how metal ions affect AA curing kinetics, offering valuable insights for optimizing adhesive formulations for industrial applications.

## 1. Introduction

Anaerobic adhesives (AAs) represent a specialized adhesive category primarily employed in metal-to-metal bonding scenarios, when the joint is in the absence of air. AAs can be defined as solvent-free, acrylic-based, one-component adhesives that cure at room temperature through a mechanism of free radicals, in the absence of air and in contact with an active metal surface. It is also possible to cure them by using an activator with metal cations and by applying heat [1].

The curing process occurs by the polymerization of the acrylic monomers triggered by an initiator. In the absence of oxygen, upon contact with an active metal surface such as iron or copper, the initiator activates, promoting polymerization, which transforms the monomers into a solid polymeric structure and results in the adhesion of the substrates. Consequently, a three-dimensional network of polymer chains is formed, strengthening and reinforcing the bonded parts. This is a targeted action that minimizes adhesive wastage and prevents premature curing in undesired areas [2,3].

The anaerobic formulation consists of a hydroperoxide as an initiator and an accelerator, which may have an oxidizing (e.g., benzoic sulfimide (saccharin)) or a reducing (e.g., tertiary amine) character. Polymerization processes occur via a radical polymerization mechanism. A single-electron transfer occurs with the formation of active RO• radicals in the presence of hydroperoxide. According to kinetic studies, polymerization occurs through redox radical polymerization. A redox reaction with metal cations generates a cumyl peroxide radical that initiates polymerization. The curing mechanisms of some AAs were studied by determining the degree of polymerization and the activation energy at various temperatures [4,5,6].

Okamoto [4,5] verified that AAs made from methyl methacrylate polymerized with o-benzoic sulfimide or with N,N-dimethyl-p-toluidine catalyzed by copper exhibit such low activation energy that they can cure at room temperature or even at lower temperatures. Additionally, depending on the polymer and amine used, the reaction rate can vary significantly. Aronovich [7] determined the reaction mechanisms using an aromatic amine (N,N-dimethyl-p-toluidine) and saccharin. B. George et al. [8,9] studied the reaction catalyzed by a transition metal in its lowest oxidation state, such as iron or copper, for the decomposition of hydroperoxide to generate active free radicals. They also found that sufficient heat energy input can also accelerate the reaction.

Various techniques have been employed to determine the kinetics of these reactions, including gas chromatography, high-performance liquid chromatography [6], and Fourier transform infrared spectroscopy (FTIR) [10,11]. To study the anaerobic curing processes in passivated stainless steels, a real-time Fourier transform infrared (RT/FT-IR) spectroscopic technique was also utilized [10]. Other characterization techniques, e.g., X-ray photoelectron spectroscopy (XPS) [12], inductively coupled plasma atomic emission spectroscopy (ICP-AES), fluorescence spectroscopy, viscosity change [13] or viscometry [8], and mechanical property change [7], were also explored to study this process. Additionally, prior to these techniques, a curing rate test was conducted in a sealed tube with a thermocouple, nitrogen atmosphere, and constant temperature bath to determine the maximum time and temperature of exothermic curing [4,5].

Since these are radical reactions, the use of initiators activated by ultraviolet radiation has also been studied [14], although in these cases at least one of the substrates must be transparent to that radiation. The curing reaction was studied by photo-DSC, applying UV radiation in the presence of oxygen. Moini et al. also used this technique to study the variation of the curing peak of solvent-resistant, bio-based anaerobic adhesives, employing glycerol-lactic acid oligomers [15]. However, to the best of the authors’ knowledge, the curing mechanism has not yet been explored using DSC. The main advantages of this technique over others, such as FTIR or XPS, are the determination of the primary reaction mechanism and the calculation of kinetic constants.

A remarkable characteristic of AAs is their rapid curing capability. Polymerization may occur within minutes once oxygen is eliminated and the adhesive is in contact with the active metal surface. It enables swift setting times, crucial in industrial applications demanding speed. The resultant post-cured polymer exhibits robust chemical and thermal properties, enduring exposure to corrosive chemicals and high temperatures. Hence, AAs find utility in harsh environments where aggressive chemicals and elevated temperatures prevail [7].

AAs show good performance in filling gaps and clearances between metal surfaces, making them suitable for bonding loosely fitted or imperfect parts. Beyond bonding, AAs serve as effective sealants, preventing liquid or gas leakage and mitigating metal surface corrosion [16]. Furthermore, despite their strength, AAs facilitate future disassembly of parts [17] when necessary. Upon applying the appropriate force and breaking the seal, the adhesive loosens, allowing component repair or replacement. The composition of the adhesive affects its rheology [12,18], thus impacting the ease of application onto the pieces to be bonded. On the other hand, enhanced adhesion can be achieved through the addition of small amounts of polar monomers, such as acrylic acid, glycidyl methacrylate, or cyanoalkyl methacrylates [19].

Regarding mechanical properties, many studies have been carried out to date. The influence of the aspect ratio (coupling length over diameter) on the shear resistance of pressed and adhesively bonded joints has been investigated [20], along with the stress distribution of cylindrical joints [21,22,23], and the effect of interference [17]. Additionally, the behavior of the adhesive in blocking nuts has also been studied [24,25].

Thermally, anaerobic adhesives show their best performance between −50 and 150 °C [26]. Regarding the durability of these joints, the aging behavior of anaerobic adhesives at high temperatures [3,27] and the effect of combined humidity and heat [28,29] have been studied.

Given these characteristics, AAs find application across diverse industrial sectors. They are utilized for thread locking on bolts and nuts, sealing joints in hydraulic and pneumatic systems, bonding machinery components, and securing automotive parts in both manufacturing and maintenance operations. The multitude of potential formulations and material substrates necessitates the study of the curing kinetics and mechanical behavior of each, ensuring the optimization of adhesive joints.

Due to the wide use of AAs in industrial applications, their composition is often modified to enhance their thermal properties and durability under high mechanical loads. At the same time, manufacturers seek to decrease toxicity and accelerate the curing rate. However, it has been observed that some new commercially available AAs do not cure completely within 24 h. For this reason, the objective of this work is to study the curing kinetics of a high-resistance AA on steel and copper substrates. The novelty of this study lies in the direct application of DSC to AAs, which has not been performed to date. Two distinct kinetic models are employed and compared, yielding crucial insights into kinetic parameters and divergent behaviors on steel and copper substrates during oxygen-free curing.

## 2. Materials and Methods

### 2.1. Materials

The adhesive used was LOCTITE^®^ 270, a liquid threadlocker engineered for a broad spectrum of metal fasteners, including stainless steel, aluminum, galvanized, and chrome-free coatings. It exhibits resilience against minor contaminations from industrial oils such as motor oils, anti-corrosion oils, and cutting fluids, and can be disassembled at 300 °C. It was supplied by Henkel Ibérica, S.A. (Barcelona, Spain) [30].

The composition of the AA includes 3,3,5-trimethylcyclohexyl methacrylate as the primary constituent and 2-2′-ethylenedioxydiethyl dimethacrylate in a lesser proportion. Additional chemical compounds comprise cumene hydroperoxide (initiator), maleic acid, 1-acetyl-2-phenylhydrazine (APH—accelerant), and traces of 1,4-naphthoquinone [31].

### 2.2. Experimental Procedure

The kinetics were investigated using thermograms obtained from a DSC 822e Mettler Toledo (Greifensee, Switzerland), employing both non-isothermal and isothermal scans. Non-isothermal scans were conducted at 5, 10, 15, and 20 °C/min from −20 to 250 °C to identify the exothermic curing peak. Approximately 4.5 mg of AA was used in an aluminum crucible with a 40 μL capacity. To facilitate anaerobic curing, AA was placed between two sheets of steel (iron surface) or copper surface with a thickness of 0.1 mm and a diameter of 6 mm inside the crucible. Nitrogen was introduced as a purge gas at a rate of 50 mL/min. Three scans for each condition were performed.

Isothermal scans were performed at 40, 60, and 80 °C for 1 h, following the non-isothermal or dynamic scan up to 250 °C. The preparation procedure for AA samples was identical for both methods. Three scans for each condition were performed.

In addition, screw torsion tests (joining screws to nuts using AA) were carried out. Torque tests were conducted on M10 × 50 carbon steel (iron ions) and yellow brass (copper ions) commercial screws at various intervals using a GOYOJO Digital Torque Wrench. Before testing, screws and nuts were cleaned with ethanol using a LT-80-PRO Ultrasonic Cleaner 1.5 L ultrasound bath (provided by Tierratech Central, Guarnizo, Spain) operating at a frequency of 37 kHz for 5 min at room temperature and then allowed to dry for one hour in a desiccator before adhesion. For each material, 48 torsion tests were carried out to determine its torsion strength at different times.

### 2.3. Thermal Analysis

Two different empirical models were used to analyze the thermograms: MFK and Kamal. Both models are based on the relationship between the variation of the enthalpy at a time t (ΔH_t_) divided by the variation of the total enthalpy (ΔH_T_) of the process (Equation (1)), namely conversion degree (α):

(1) $$\alpha \;=\frac{\Delta H_{t}}{\Delta H_{T}}$$

#### 2.3.1. Model-Free Kinetics (MFK)

The MFK model utilized in this study was implemented on non-isothermal thermograms. For this purpose, the STARe Evaluation V12.10 software provided by Mettler Toledo was employed. This model, a blend of empirical and mathematical formulations, derives its equations from the works of Vyazovkin and Wight [32]. The analysis proceeds through the following steps: (1) determination of α from non-isothermal curves, (2) calculation of activation energy (E_a_) as a function of α, and (3) generation of isothermal curves at various temperatures based on the results of activation energy, presenting an alternative approach to iso-conversional methods [33].

This model is typically applied in epoxy adhesives, but can be applied in any process in which an exothermic or endothermic reaction occurs, such as polymerization or curing [34] and decomposition [35].

#### 2.3.2. Kamal’s Model

The thermogram data under isothermal conditions were subjected to fitting, employing a model tailored for heterogeneous reactions, using two kinetic equations known as autocatalytic or nth-order kinetics. Autocatalytic mechanisms are distinguished by exhibiting a peak reaction rate occurring at around 30–40% of the curing process. It is worth noting that in many cases, the kinetic parameters within these models lack a direct physicochemical interpretation and are treated as adjustment parameters. Kamal’s method [36] was employed to compute the kinetic parameters as per Equation (2), which encompasses both autocatalytic and nth-order mechanisms:

(2) $$\frac{\partial \alpha }{\partial t}=\left(k_{1}+{\;k}_{2}\alpha^{m}\right)\;{\left(1-\alpha \right)}^{n}$$

where t represents time, k_1_ and k_2_ are the rate constants of the nth-order and autocatalytic reaction, respectively, and n and m represent the reaction order. Kinetic parameters were obtained by iteration of Equation (2) with the software Origin V6.0.

In this equation, α is calculated according to Equation (3), which represents a small variation of Equation (1). Here, the variation of the isothermal enthalpy at a time t is divided by the variation of the total enthalpy calculated as the sum of both isothermal and dynamic scans.

(3) $$\alpha =\frac{\Delta H_{{\mathrm{Isothermal}}_{t}}}{\Delta H_{\mathrm{Isothermal}}+\Delta H_{\mathrm{Dynamic}}}$$

Once the reaction constants at different temperatures were calculated, the activation energy (E_a_) of the curing process was calculated using an Arrhenius-type equation (Equation (4)).

(4) $$\mathrm{Ln}\;k=\mathrm{Ln}\;A-\frac{E_{a}}{R\;T}\;$$

where k is the reaction constant (k_1_ or k_2_) that provides an idea of the reaction rate, A is a constant that depends on the radicals or ions involved in the reaction, R is the gas constant (8.341 J/mol K), and T is the absolute temperature.

## 3. Results

### 3.1. MFK Model

#### 3.1.1. MFK Model

**Figure 1:**
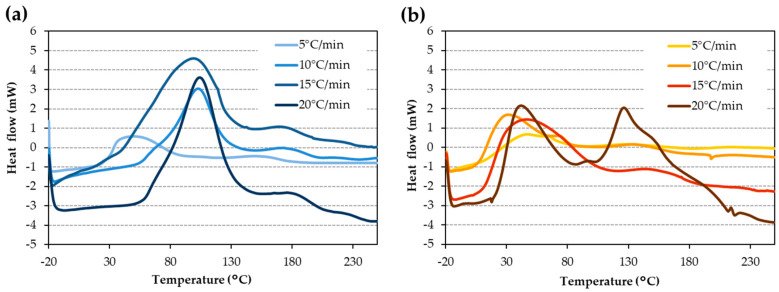
Thermograms at different rates for (**a**) iron surface and (**b**) copper surface.

**Figure 2:**
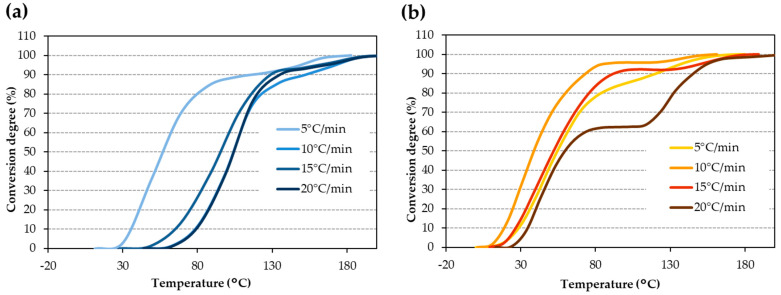
Conversion degree at different rates for (**a**) iron surface and (**b**) copper surface.

The non-isothermal thermograms acquired by DSC for the iron surface (Figure 1a) and the copper surface (Figure 1b) at four distinct rates were examined. The differentiated average of the four curves with respect to time provides the enthalpy of the curing process on iron and copper surfaces, which is 199 ± 2 Jg^−1^ and 146 ± 2 Jg^−1^, respectively. The thermograms had two peaks, which were more evident for high rates. Conversion degree or alpha (α) value vs. temperature was determined for the iron surface (Figure 2a) and the copper surface (Figure 2b). In these curves, the double peak observed in the thermograms at the highest heating rate appears as a change in the slope, like a shoulder.

**Figure 3:**
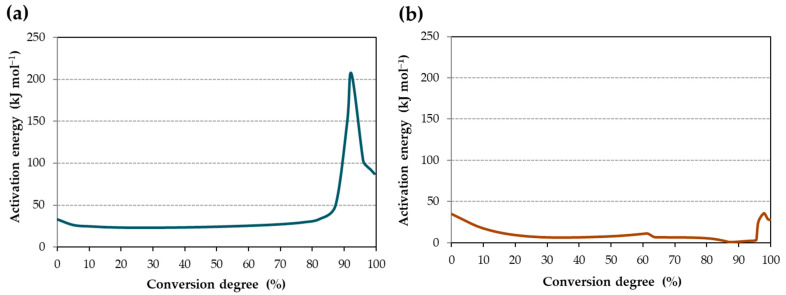
Activation energy for (**a**) iron surface and (**b**) copper surface.

From conversion degree vs. temperature curves, the activation energy (E_a_) of the curing reaction was obtained by the software for the iron surface (Figure 3a) and the copper surface (Figure 3b). The increment of E_a_ for high α is due to the curing mechanism, which occurs in two stages. Subsequently, the E_a_ values were employed to simulate isothermal processes at various temperatures (Table 1).

In the simulation, the difference in curing times for the iron surface (Table 1(a)) and the copper surface (Table 1(b)) can be observed. The adhesive cured on an iron surface requires significantly longer times compared to when it is cured on a copper surface. The effect of the polymerization degree on the time needed to continue the process is also evident in the simulation. From 90% curing onwards, much more time is needed to complete the curing of the AA. This increase in time is more pronounced for the iron surface (Table 1(a)) than for the copper surface (Table 1(b)).

#### 3.1.2. Kamal’s Model

**Figure 4:**
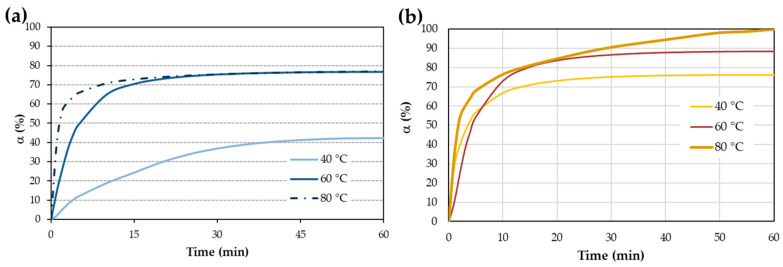
Conversion degree at different temperatures for (**a**) iron surface and (**b**) copper surface.

The total enthalpies calculated by isothermal and dynamic scans were 195 ± 1 Jg^−1^ for iron and 146 ± 5 Jg^−1^ for copper. These values are similar to those calculated from non-isothermal scans. Figure 4 shows α vs. time for both metals at different temperatures, with maximum values of 42% and 77% for the iron surface and 76%, 89%, and 100% for the copper surface. As expected, α increases with temperature; however, the same curing degree was found for the iron surface at 60 °C and 80 °C, over 25% of α (Figure 4a). On surfaces with copper ions (Figure 4b), the reaction is slightly faster at 40 °C, but this is only up to 5% conversion, which falls within the model’s error margin at the beginning and end of the process.

**Figure 5:**
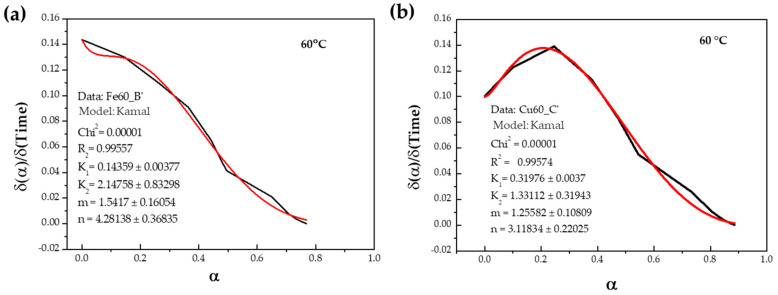
Calculation of kinetic parameters at 60 °C for (**a**) iron surface and (**b**) copper surface. The black line corresponds to the δα/δt vs. α, and the red line represents the iteration of Equation (2) to calculate the parameters.

Kamal’s model is based on two mechanisms: nth-order and autocatalytic. The parameters were calculated according to Equation (2) by iteration of α derivation with respect to time vs. α. In Figure 5, these fittings for the iron surface (Figure 5a) and the copper surface (Figure 5b) at 60 °C can be observed. Table 2 shows the kinetic parameters at three temperatures for iron and copper surfaces.

**Figure 6:**
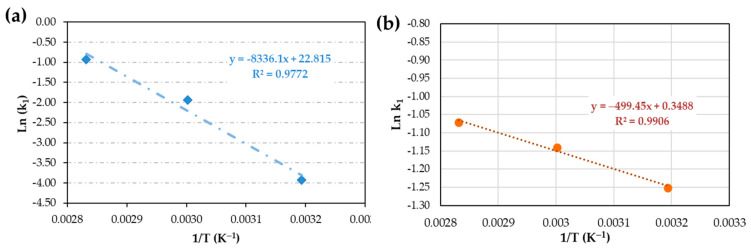
Activation energy for the anaerobic curing for (**a**) an iron surface and (**b**) a copper surface.

Reaction constant k_1_ is lower than k_2_; thus, k_1_ corresponds to the slowest reaction and E_a_ values depend on this constant. When Equation (4) is applied (Figure 6), from the slope of the line, E_a_ is calculated. E_a_ for iron surface is 69.5 kJ/mol and for copper surface is 4.2 kJ/mol, which corresponds to a slower reaction and less efficacy of iron ions in the curing process.

#### 3.1.3. MFK vs. Kamal

**Figure 7:**
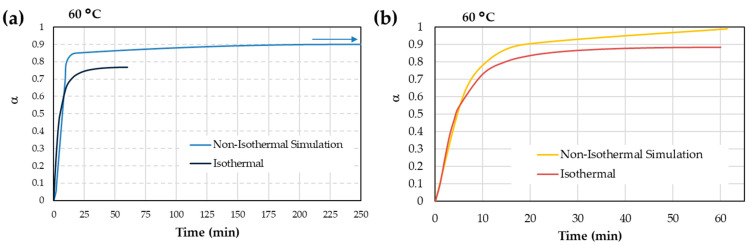
Comparison of kinetic models at 60 °C (**a**) for iron surface and (**b**) for copper surface. The arrow means curing process is not finished.

In Figure 7a, an analysis is conducted of isothermal curing at 60 °C alongside simulation results from the MFK method performed at the same temperature. An isothermal scan was performed for one hour, and it was found that AA achieved a curing degree of 77%. In contrast, an MFK simulation of the same duration predicts an 86% curing degree, showing an 11.7 % deviation.

The same analysis as that carried out for iron surfaces was performed for a copper surface. Figure 7b shows the isothermal curing process and the MFK simulation at 60 °C. After one hour of curing, the isothermal scan showed an 89% curing degree, whilst the MFK simulation predicts a complete 100% curing in one hour, representing a 12.4% error in the simulation compared to the experimental calculation.

### 3.2. Mechanical Testing

**Figure 8:**
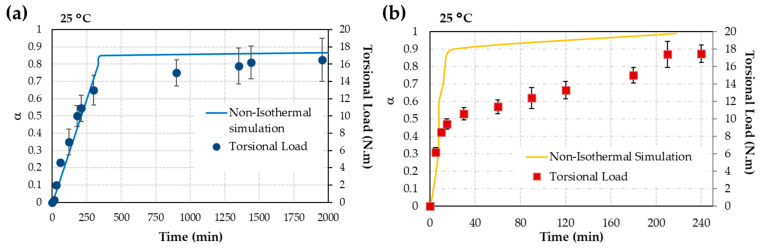
Comparison between MFK simulation and torsional load at 25 °C (**a**) for iron surface and (**b**) for copper surface.

Moving to Figure 8a, for the iron surface, the MFK simulation is juxtaposed with torsional load at 25 °C. Throughout the 33 h of test duration, the curing fluctuated between 85% at 6 h and 87% at 143 h, yielding a screw resistance of 16 N·m. It could be concluded that the torsional load depends directly on the curing degree. The maximum of both parameters was achieved after 6 h, remaining constant until the end of the test.

In Figure 8b, the MFK simulation is compared with torsional strength at 25 °C, over a duration of up to 240 min (roughly 4 h), for the copper surface. At the 208 min mark, the curing reaches 100%, resulting in a screw resistance of 17.4 N·m, with similar degrees of resistance observed at longer intervals. In this case, the relation between curing degree and torsional load is not direct, as in the case of the iron surface. The achievement of resistance is slightly delayed with respect to the curing degree.

## 4. Discussion

### 4.1. Curing Mechanism of AA

**Figure 9:**
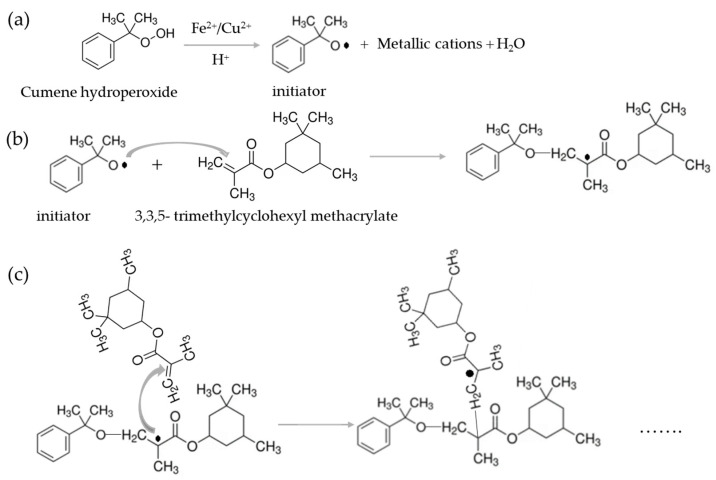
(**a**) The formation of the cumene hydroperoxide radical; (**b**) the reaction of the initiator radical with the monomer 3,3,5-trimethylcyclohexyl methacrylate to initiate the curing reaction; and (**c**) the progression of the curing reaction.

**Figure 10:**
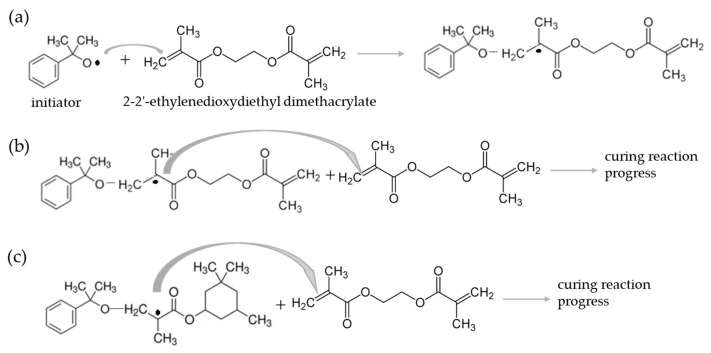
(**a**) A reaction of the initiator radical with the monomer 2-2′-ethylenedioxydiethyl dimethacrylate to initiate the curing reaction (**b**) and the progress of the curing reaction; (**c**) a reaction of the radical of 3,3,5-trimethylcyclohexyl methacrylate with 2-2′-ethylenedioxydiethyl dimethacrylate to initiate the curing reaction of the other different polymer.

The AA used in this work shows two differentiated curing peaks in the dynamic scans of DSC (Figure 1) due to the presence of two monomers that do not cure simultaneously. From the area of the peaks, it could be concluded that the monomer in the highest concentration polymerizes at a lower temperature. In an acid medium and the presence of metallic cations, cumene hydroperoxide acts as the initiator and provides free radicals by a redox process (Figure 9a). These radicals react with the major monomer (3,3,5-trimethylcyclohexyl methacrylate), initiating the curing reaction (Figure 9b). The reaction progresses (Figure 9c) by further monomer addition, forming a cross-linked structure [37]. This would be the case if there were only one monomer. However, the minor monomer (2-2′-ethylenedioxydiethyl dimethacrylate) reacts with the remaining initiator, creating a cross-linked network (Figure 10a). The reaction of the second monomer may continue by cross-linking with itself (Figure 10b), or with the remaining radicals from the first monomer (Figure 10c). The polymerization of both monomers can occur simultaneously or in two different stages.

Therefore, three different radicals coexist during the curing process, and they can combine randomly, resulting in different adhesive properties in terms of viscosity and/or curing. Consequently, the curing degree influences the mechanical properties of the adhesive joint.

### 4.2. Comparison between Empirical Kinetic Models

#### 4.2.1. MFK Model

The presence of iron or copper metal ions influences the curing process. In this work, it was found that the curing enthalpy is higher on an iron surface compared to a copper surface by approximately 27% (Figure 1). Additionally, it was confirmed that the four scans exhibit the same enthalpy, a logical and necessary condition for validating the process considering the previously explained randomness of the polymerization process. Furthermore, the heating rate (expressed in °C/min) affects the curing time: the higher the rate, the shorter the curing time.

The activation energy (E_a_) exhibits slight variations in the degree of conversion (Figure 3) and can be segmented into three phases: initiation, propagation, and termination. Initially, for iron (Figure 3a), 33 kJ/mol is required, a relatively low energy demand when an initiator is present in the reaction. This requirement remains consistent from approximately 10% to 70% conversion, hovering around 24 kJ/mol. Towards the end, the E_a_ gradually escalates, reaching a peak of 207 kJ/mol at 85–90% conversion. This end-stage peak aligns with the curing process of certain acrylates, where a scarcity of free radicals or steric hindrance may make the curing process difficult [38].

In contrast, the presence of copper ions exhibits no significant influence on the initiation phase of the reaction (Figure 3b), with E_a_ values similar to those observed on the iron surface. However, from 20% to 85% conversion, the E_a_ diminishes significantly to 7 kJ/mol, indicating a notable catalytic effect of copper ions on the reaction progress. This may be because the reaction is ending and there are few radicals left from the monomer to continue until those from the minority monomer begin to form, which rises again to 98 kJ/mol for the final curing process. As in the previous case of the iron, the activation energy rises to 207 kJ/mol at the end of the curing process. This increase in E_a_ may be attributed to the curing of the second acrylate or to the challenge of locating the remaining free radicals of the main monomer, which is still uncured.

The simulation based on E_a_ logically reflects the curing process of the two monomers (Table 1). Temperature significantly influences reaction kinetics, leading to notable differences between iron ions (Table 1(a)) and copper ions (Table 1(b)). In the curing of the AA on an iron surface (Table 1(a)), a pronounced increase in curing time is evident between 85% and 90% conversion, but this increase is even more significant between 90% and 92%, reaching 237 days to achieve 99% conversion at 25 °C. Similarly, at 80 °C, these time increments between 85–90% and 90–92% are observed, but to a lesser extent, with curing completed within one day.

In contrast, the copper surface (Table 1(b)) exhibits much faster curing rates, with no abrupt changes in time observed. Curing is achieved in 218 min (less than four hours) at 25 °C and in 30 min at 80 °C. This faster curing rate on copper than on iron was also found by other researchers with spectroscopy [39].

#### 4.2.2. Kamal’s Model

Kamal’s model, utilizing the isothermal method, yields curing enthalpies of 195 ± 1 J/g for iron and 146 ± 5 J/g for copper. Due to the isothermal process lasting for 1 h, complete curing is not achieved at any temperature for steel (Figure 4a), with percentages of 42%, 77%, and 77% observed for the three temperatures used (40 °C, 60 °C, and 80 °C, respectively). The iteration produces an nth-order type curve for steel, despite the full iteration of Kamal’s equation (Figure 5a). The calculated activation energy (E_a_) for the entire process, as per Equation (4), is 69.5 kJ/mol (from Figure 6a). When working with epoxy adhesives, the E_a_ value at 50% conversion coincides quite well with the E_a_ calculated from Kamal’s method. However, in this case, it does not coincide, being almost one-third of that obtained by MFK. This discrepancy may be due to a different curing mechanism in AAs.

Thermograms for AA on copper surface exhibit complete curing of 100% at 80 °C. At 40 °C and 60 °C, curing percentages are 76% and 86%, respectively (Figure 4b). Iteration curve shapes also align with the nth-order type (Figure 5b), with E_a_ values notably low (Figure 6b) at 4.2 kJ/mol. This value seems to be more in agreement with that observed by the MFK method at 50% conversion (6 kJ/mol). Lower E_a_ means that it is easier for the reaction to occur.

In both cases, iteration for the calculation of kinetic parameters (Table 2) demonstrates high accuracy, with R^2^ values close to 1 for all temperatures. Only in the case of the iron surface at 40 °C, R^2^ drops slightly to 0.96, which is still an acceptable value. Reaction constants increase with temperature, indicating accelerated reaction rates due to enhanced monomer or radical movement, facilitating their contact and reaction. The slowest reaction constant corresponds to k_1_, hence E_a_ is calculated based on k_1_.

The adjustments made to Kamal’s equation (Equation (2)) do not provide sufficient information about the curing mechanism, as it seems that two mechanisms are not involved; rather, there is a single mechanism, in which perhaps the diffusion process (Equation (5)) should be included. This could provide more information about high-temperature curing. However, the only equation that accounts for diffusion is Kamal’s own equation, to which an additional term is added for a better fit of the iteration curve to the experimental data [40].

(5) $$\frac{\partial \alpha }{\partial t}=\left(k_{1}+{\;k}_{2}\alpha^{m}\right)\;{\left(1-\alpha \right)}^{n}\;F\left(\alpha \right)$$

F(α) can be computed as F(α) = 1/1 + e^C(α−αc)^, where C is the diffusion constant and α_c_ the critical conversion degree value.

#### 4.2.3. Comparison between MFK and Kamal’s Models

The comparison between the two kinetic methods suggests that the MFK method offers considerable reliability, as it provides more comprehensive information about the entire curing process. The MFK method concurs with the curing of two acrylates not occurring simultaneously. However, when comparing the two methods (Figure 7a,b), Kamal’s method appears to be more conservative at short times (1 h), yielding lower degrees of conversion (around 12% of difference between MFK and Kamal calculations). This is feasible considering that the MFK data are a simulation, while the Kamal data are taken directly from thermograms.

The most significant information obtained from Kamal’s method are the reaction constants (Table 2), where the k_1_ value is found to be lower than k_2_, thereby governing the reaction. Additionally, k_1_ is observed to be higher for a copper surface than for an iron surface at the two lowest temperatures, indicating a faster reaction on copper surfaces.

### 4.3. A comparison of MFK Simulation with Torsion Tests

In relation to the screws, the maximum torsional load is not reached for either iron or copper ions. In the case of iron ions, the curing process is very long, and the maximum curing is not reached, which could give a torque of around 33 N·m, as per the manufacturer’s datasheet. The copper ions also do not have very high resistance, perhaps due to the surface oxidation of zinc; consequently, copper cations were not easily accessible for the redox reaction of the formation of initial free radicals to occur. However, the torsion strength curves do follow a relationship with the simulation at 25 °C of the AA curing process by MFK (Figure 8a,b). Ultimately, the torsional load of the screws with the nut was similar, but it was reached at different times, with the reaction being faster when copper ions were present compared to iron ions. For this reason, the activation energy of the curing process is lower when copper ions are present. Since the torsional load is similar in both cases, the state of the adhesive would be the same.

### 4.4. Potential Causes That Affect Curing

When explaining the influence of metal surfaces on the curing of anaerobic composites, it is assumed that metal oxide compounds on the surface interact with the acidic agent of the accelerator system to form soluble salts, which break down hydroperoxides via a radical mechanism. The main influence of transition metal ions is from the lower oxidation state [41]. Cu^2+^ can be reduced to Cu^1+^ by hydroperoxide, while Fe^3+^ is not reduced to Fe^2+^ [42]. It is suggested that Fe^3+^ is reduced to Fe^2+^ with the help of dimethylparatoluidine, sustaining the redox reaction [43]. According to the product safety sheet [31], this AA does not contain this component. This may be the cause of the slower curing of the AA used on iron surfaces. XPS analyses carried out on copper surfaces have detected the presence of both copper cations [44]. Faster curing can be achieved by treating one or both surfaces with a primer. These primers are usually based on dilute solutions of transition metal salts in a volatile organic solvent [19].

It is known, through word of mouth, that screws and nuts are coated with a protective oil that may contain certain inorganic nitrite compounds. Even after removing the oil, traces of nitrites often remain, which can prevent the curing of anaerobic adhesives. This phenomenon could also account for the variability in results and the low torsional load values obtained. Consequently, an effective nitrite cleaner would be necessary at the user level to achieve maximum loads.

## 5. Conclusions

The results of this work highlight the significant influence of metal ions on the curing kinetics of anaerobic adhesives, providing insights for optimizing adhesive performance based on substrate material. To analyze this influence, two kinetic models were used to study the curing of an anaerobic adhesive via DSC: model-free kinetics (MFK) for non-isothermal scans and the Kamal model for isothermal scans followed by non-isothermal scans.

Both models provided the same enthalpy of curing, with higher ΔH for steel (196 J/g) compared to copper (146 J/g). In addition, the activation energy of the curing process also varies between iron and copper ions. Curing on copper ions occurs rapidly, reaching maximum curing at room temperature in less than 4 h, while curing on iron surfaces remains incomplete.

The MFK method revealed that the two acrylates in the AA formulation do not cure simultaneously, providing Ea in relation to the degree of conversion (α).

The Kamal method identified the rate constants, concluding that an nth-order reaction controls the curing process. This method also confirmed that curing on iron ions is slower than on copper ions, aligning with the MFK analysis.

Finally, in terms of mechanical resistance, the torsional load tests showed that neither iron nor copper ions reached the maximum resistance.

## Figures and Tables

**Table 1:** A simulation of the isothermal process at different temperatures: (a) an iron surface and (b) a copper surface.

(a) Applied Kinetics: Conversion					(b) Applied Kinetics: Conversion				
α (%)	Time (min)				α (%)	Time (min)			
	Temperature (°C)					Temperature (°C)			
	25	40	60	80		25	40	60	80
5	24	6	2	1	5	2	1	1	0
10	44	10	2	1	10	3	2	2	1
20	86	18	4	2	20	6	5	4	3
30	127	26	5	3	30	7	7	6	5
40	168	33	6	3	40	8	7	6	5
50	210	41	7	3	50	8	7	6	5
60	251	49	8	4	60	8	7	6	5
70	292	57	9	5	70	12	10	9	8
80	334	65	10	6	80	14	12	11	10
85	354	69	18	7	85	17	14	12	11
90	8613	1624	223	38	90	21	20	19	18
92	312,470	65,580	9192	1609	92	174	80	35	20
99	340,620	65,630	9199	1611	99	218	126	61	30

**Table 2:** Kinetic parameters (Equation (2)) at different temperatures.

	Iron Surface Temperature (°C)			Copper Surface Temperature (°C)		
	40	60	80	40	60	80
Chi^2^	5.2 × 10^−6^	1.0 × 10^−5^	1.1 × 10^−4^	3.0 × 10^−5^	1.0 × 10^−5^	1.5 × 10^−4^
R^2^	0.95733	0.99557	0.99311	0.99663	0.99574	0.99825
k_1_ (min^−1^)	0.01965	0.14359	0.39437	0.28626	0.31976	0.34273
k_2_ (min^−1^)	0.04992	2.14758	14.21652	0.42436	1.33112	33.13826
m	0.38327	1.5417	1.40924	0.00066	1.25582	2.37392
n	5.79129	4.28138	5.37868	3.85571	3.11834	5.69449

## Data Availability

The data files from the equipment utilized, along with the Excel or Origin files used for calculations, are available upon specific request to any researchers or reviewers wishing to consult them.

## References

[1] Baldwin T.R. (1986). Anaerobic Adhesives. Mater. Sci. Technol..

[2] Klemarczyk P., Guthrie J. (2010). 5—Advances in Anaerobic and Cyanoacrylate Adhesives. Advances in Structural Adhesive Bonding.

[3] Maggione S., Baena M.D., Stagnaro P., Giorgio L. (2021). A Review of Structural Adhesive Joints in Hybrid Joining Processes. Polymers.

[4] Okamoto Y. (1990). Anaerobic Adhesive Cure Mechanism—I. J. Adhes..

[5] Okamoto Y. (1990). Anaerobic Adhesive Cure Mechanism—II. J. Adhes..

[6] Raftery D., Smyth M.R., Leonard R.G., Heatley D. (1997). Effect of Copper(II) and Iron(III) Ions on Reactions Undergone by the Accelerator 1-Acetyl-2-Phenylhydrazine Commonly Used in Anaerobic Adhesives. Int. J. Adhes. Adhes..

[7] Aronovich D.A. (2021). Achievements in Improving Thermal Properties of Anaerobic Adhesives. Review. Polym. Sci.-Ser. D.

[8] George B., Grohens Y., Touyeras F., Vebrel J. (2000). New Elements for the Understanding of the Anaerobic Adhesives Reactivity. Int. J. Adhes. Adhes..

[9] George B., Grohens Y., Touyeras F., Vebrel J. (1998). Calorimetric Investigation of Autoacceleration in the Metal-Catalysed Cure of Anaerobic Adhesives. J. Adhes. Sci. Technol..

[10] Yang D.B., Wolf D., Wakamatsu T., Holmes M. (1995). Characterization of Cure Profiles of Anaerobic Adhesives by Real-Time FT-IR Spectroscopy. Part II. Surface Activation. J. Adhes. Sci. Technol..

[11] Sineokov A.P., Aronovich D.A., Murokh A.F., Khamidulova Z.S. (2008). Mechanism of Initiation of the Curing of Anaerobic Adhesives. Int. Polym. Sci. Technol..

[12] Maandi E., Sung C.S.P. (2008). In Situ Fluorescence Spectroscopic Studies of Polymerization of Anaerobic Adhesives. J. Appl. Polym. Sci..

[13] Madrid M., Martínez M.A., Garriga A. (2004). Rheological Behavior of Anaerobic Adhesives: Rheological Profile Modelling Depending on the Composition. J. Adhes. Sci. Technol..

[14] Aronovich D.A., Sineokova O.A., Zaitova N.V., Khamidulova Z.S., Vinokurova N.I., Lyapishev V.M. (2015). UV-Curable Anaerobic Adhesive Compositions. Polym. Sci.-Ser. D.

[15] Moini N., Khaghanipour M., Kabiri K., Salimi A., Zohuriaan-Mehr M.J., Jahandideh A. (2019). Engineered Green Adhesives Based on Demands: Star-Shaped Glycerol-Lactic Acid Oligomers in Anaerobic Adhesives. ACS Sustain. Chem. Eng..

[16] Dunn D. (2010). Update on Engineering and Structural Adhesives.

[17] Croccolo D., De Agostinis M., Fini S., Olmi G., Paiardini L., Robusto F. (2020). Influence of the Interference Level and of the Assembly Process on the Shear Strength of Loctite 648 Anaerobic Adhesive. J. Adhes..

[18] Madrid M., González-Gutiérrez L., Martínez M.A., Garriga A. (2006). Modeling the Rheology of Anaerobic Adhesive Formulations. J. Adhes. Sci. Technol..

[19] Stamper D.J. (1983). Curing Characteristics of Anaerobic Sealants and Adhesives. Br. Polym. J..

[20] Martínez M.A., Pantoja M., Abenojar J., Velasco F., Durbán M. (2007). Analysis of Shear Strength of Cylindrical Assemblies with Anaerobic Adhesives Using Weibull Statistics. J. Adhes. Sci. Technol..

[21] Lidón J., Pérez B., Martínez M.A., Madrid M. (2005). Calculation of the Strength of Cylindrical Assemblies with an Anaerobic Adhesive. J. Adhes. Sci. Technol..

[22] Corigliano P., Ragni M., Castagnetti D., Crupi V., Dragoni E., Guglielmino E. (2021). Measuring the Static Shear Strength of Anaerobic Adhesives in Finite Thickness under High Pressure. J. Adhes..

[23] Castagnetti D., Corigliano P., Barone C., Crupi V., Dragoni E., Guglielmino E. (2022). Predicting the Macroscopic Shear Strength of Tightened-Bonded Joints from the Intrinsic High-Pressure Properties of Anaerobic Adhesives. Metals.

[24] Dragoni E., Mauri P. (2000). Intrinsic Static Strength of Friction Interfaces Augmented with Anaerobic Adhesives. Int. J. Adhes. Adhes..

[25] Dragoni E., Mauri P. (2002). Cumulative Static Strength of Tightened Joints Bonded with Anaerobic Adhesives. Proc. Inst. Mech. Eng. Part L J. Mater. Des. Appl..

[26] Petrova A.P., Lukina N.F. (2008). Adhesive Technologies in Aircraft Construction. Polym. Sci. Ser. D.

[27] Cherry B.W., Ye Y.Q. (1992). The Behaviour of High Temperature Anaerobic Adhesives. Int. J. Adhes. Adhes..

[28] Croccolo G., de Agostinis M., Fini S., Olmi G., Paiardini L., Robusto F. (2022). Effects of Aging Temperature and Humidity on the Response of Medium and High Strength Threadlockers. J. Adhes..

[29] Sakai K., Nassar S.A. (2017). Failure Analysis of Composite-Based Lightweight Multimaterial Joints in Tensile-Shear Tests after Cyclic Heat at High-Relative Humidity. J. Manuf. Sci. Eng. Trans. ASME.

[30] Henkel Iberica S.A. Loctite ® 270TM Technical Data Sheet 2019. https://www.henkel-adhesives.com/es/en/product/threadlockers/loctite_2702.html.

[31] Henkel Iberica S.A. Loctite ® 270TM Safety Data Sheet According to Regulation (EC) No. 1907/2006 in Its Updated Version 2024. https://mysds.henkel.com>SAP>DocCocContentData.pdf.

[32] Vyazovkin S., Wight C.A. (1999). Model-Free and Model-Fitting Approaches to Kinetic Analysis of Isothermal and Nonisothermal Data. Thermochim. Acta.

[33] Sewry J.D., Brown M.E. (2002). “Model-Free” Kinetic Analysis?. Thermochim. Acta.

[34] Abenojar J., Lopez de Armentia S., Barbosa A.Q., Martinez M.A., del Real J.C., da Silva L.F.M., Velasco F. (2023). Magnetic Cork Particles as Reinforcement in an Epoxy Resin: Effect of Size and Amount on Thermal Properties. J. Therm. Anal. Calorim..

[35] Abenojar J., Aparicio G.M., Butenegro J.A., Bharami M., Martínez M.A. (2024). Decomposition Kinetics and Lifetime Estimation on Natural Fibre Reinforced Thermoplastic Composites. Materials.

[36] Kamal M.R., Sourour S. (1973). Kinetics and Thermal Characterization of Thermoset Cure. Polym. Eng. Sci..

[37] Lai P.L., Chen L.H., Chen W.J., Chu I.M. (2013). Chemical and Physical Properties of Bone Cement for Vertebroplasty. Biomed. J..

[38] Ma T., Ma J., Zhang J., Cheng J., Yang C. (2020). Curing Behaviors and Properties of Epoxy Resins with Para-Hexatomic Ring Blocks: Excellent Comprehensive Performances of Tetrafluorophenyl. Polymer.

[39] George B., Touyeras F., Grohens Y., Vebrel J. (1997). Spectroscopic and Mechanical Evidence of the Influence of the Substrate on an Anaerobic Adhesive Cure. Int. J. Adhes. Adhes..

[40] Barbosa A.Q., Da Silva L.F.M., Abenojar J., Del Real J.C., Paiva R.M.M., Öchsner A. (2015). Kinetic Analysis and Characterization of an Epoxy/Cork Adhesive. Thermochim. Acta.

[41] Moane S., Raftery D.P., Smyth M.R., Leonard R.G. (1999). Decomposition of Peroxides by Transition Metal Ions in Anaerobic Adhesive Cure Chemistry. Int. J. Adhes. Adhes..

[42] Boeder C., Hartshorn S. (1986). Anaerobic and Structural Acrylic Adhesives. Chemistry and Technology.

[43] Lees W.A. (1979). The Science of Acrylic Adhesives. Br. Polym. J..

[44] Hudak S.J., Boerio F.J., Clark P.J., Okamoto Y. (1990). XPS Analysis of the Interphase between an Anaerobic Adhesive and Metal Substrates. Surf. Interface Anal..

